# BACE1 Inhibition Induces a Specific Cerebrospinal Fluid β-Amyloid Pattern That Identifies Drug Effects in the Central Nervous System

**DOI:** 10.1371/journal.pone.0031084

**Published:** 2012-02-06

**Authors:** Niklas Mattsson, Lawrence Rajendran, Henrik Zetterberg, Mikael Gustavsson, Ulf Andreasson, Maria Olsson, Gunnar Brinkmalm, Johan Lundkvist, Laura H. Jacobson, Ludovic Perrot, Ulf Neumann, Herman Borghys, Marc Mercken, Deborah Dhuyvetter, Fredrik Jeppsson, Kaj Blennow, Erik Portelius

**Affiliations:** 1 Clinical Neurochemistry Laboratory, Department of Psychiatry and Neurochemistry, Institute of Neuroscience and Physiology, The Sahlgrenska Academy, University of Gothenburg, Mölndal, Sweden; 2 Systems and Cell Biology of Neurodegeneration, Division of Psychiatry Research, University of Zurich, Zurich, Switzerland; 3 Innovative Medicines, Central Nervous System and Pain iMed, Department of Neuroscience, AstraZeneca R&D, Södertälje, Sweden; 4 Neuroscience Discovery, Novartis Institutes for BioMedical Research, Basel, Switzerland; 5 Neuroscience Therapeutic Area, Janssen Research and Development, Beerse, Belgium; Nathan Kline Institute and New York University School of Medicine, United States of America

## Abstract

BACE1 is a key enzyme for amyloid-β (Aβ) production, and an attractive therapeutic target in Alzheimer's disease (AD). Here we report that BACE1 inhibitors have distinct effects on neuronal Aβ metabolism, inducing a unique pattern of secreted Aβ peptides, analyzed in cell media from amyloid precursor protein (APP) transfected cells and in cerebrospinal fluid (CSF) from dogs by immunoprecipitation-mass spectrometry, using several different BACE1 inhibitors. Besides the expected reductions in Aβ1-40 and Aβ1-42, treatment also changed the relative levels of several other Aβ isoforms. In particular Aβ1-34 decreased, while Aβ5-40 increased, and these changes were more sensitive to BACE1 inhibition than the changes in Aβ1-40 and Aβ1-42. The effects on Aβ5-40 indicate the presence of a BACE1 independent pathway of APP degradation. The described CSF Aβ pattern may be used as a pharmacodynamic fingerprint to detect biochemical effects of BACE1-therapies in clinical trials, which might accelerate development of novel therapies.

## Introduction

Alzheimer's disease (AD) is the most common neurodegenerative disease world-wide [1]. Accumulation of toxic amyloid-β (Aβ) peptides is thought to be at the core of AD pathogenesis [2]–[4]. Hence, one of the main targets of novel disease-modifying drugs is to minimize the brain Aβ load by targeting the β- and γ-secretases that cleave the amyloid precursor protein (APP) to generate Aβ [5]. β-Secretase has been identified as the membrane-anchored aspartyl protease β-site APP-cleaving enzyme 1 (BACE1, also called Asp2 and memapsin2) [6]–[8]. BACE1 inhibitors are recognized as potential candidates for disease-modifying AD drugs, but their development has been unsatisfactory to date, due to difficulties identifying compounds with desired effects in the central nervous system (CNS), especially due to difficulties in achieving blood-brain barrier penetration [5], [9]. Markers of biochemical drug effects *in vivo* - so called theragnostic or pharmacodynamic biomarkers - could identify effective compounds and facilitate drug development [10]. Analysis of Aβ isoforms in the cerebrospinal fluid (CSF) is a potentially informative measure of APP metabolism occurring in the brain. We tested here the hypothesis that a distinct Aβ peptide pattern can be used to identify effects of BACE1 inhibition in mammals, by analyses in cell media and in dog CSF. Several Aβ isoforms exist *in vivo*, depending on different degradation pathways of APP [11]. Aβ in plaques consists of up to 43 amino acids, with Aβ4-42 and Aβ1-42 being among the most abundant [12]–[14]. In contrast, the Aβ pattern in CSF is dominated by Aβ1-40 and several C-terminally truncated isoforms ranging down to Aβ1-13, with Aβ1-42 present only in small amounts [15], [16]. The membrane-bound γ-secretase complex is directly or indirectly involved in the APP processing that produces the different C-terminal endings of Aβ in the range Aβ1-17 to Aβ1-42 [17], [18] while BACE1 mediates the cleavages at both the N-terminal and at Glu11 of Aβ to produce Aβ1-X and Aβ11-X isoforms [7]. APP may also be cut by α-secretase enzymes within the Aβ domain [19], [20], and α- and β-secretase pathways may converge to produce short Aβ peptides, including Aβ1-14, Aβ1-15 and Aβ1-16 [17], [18].

We show here that BACE1 inhibition results in a distinctly altered CSF Aβ pattern, including reduced levels of Aβ1-34 and increased levels of Aβ5-40, besides the expected reduced levels of Aβ1-40 and Aβ1-42. The Aβ5-40/Aβ1-34 ratio was highly elevated in the CSF of treated animals and clearly distinguished active treatment from placebo. This Aβ pattern may be useful as a specific and sensitive pharmacodynamic fingerprint of BACE1 inhibition to assess *in vivo* biochemical effects in CNS in clinical trials of BACE1 inhibitors and thereby accelerate drug development.

## Results

### BACE1-inhibition induces a specific Aβ peptide pattern in cell media

**Figure 1:**
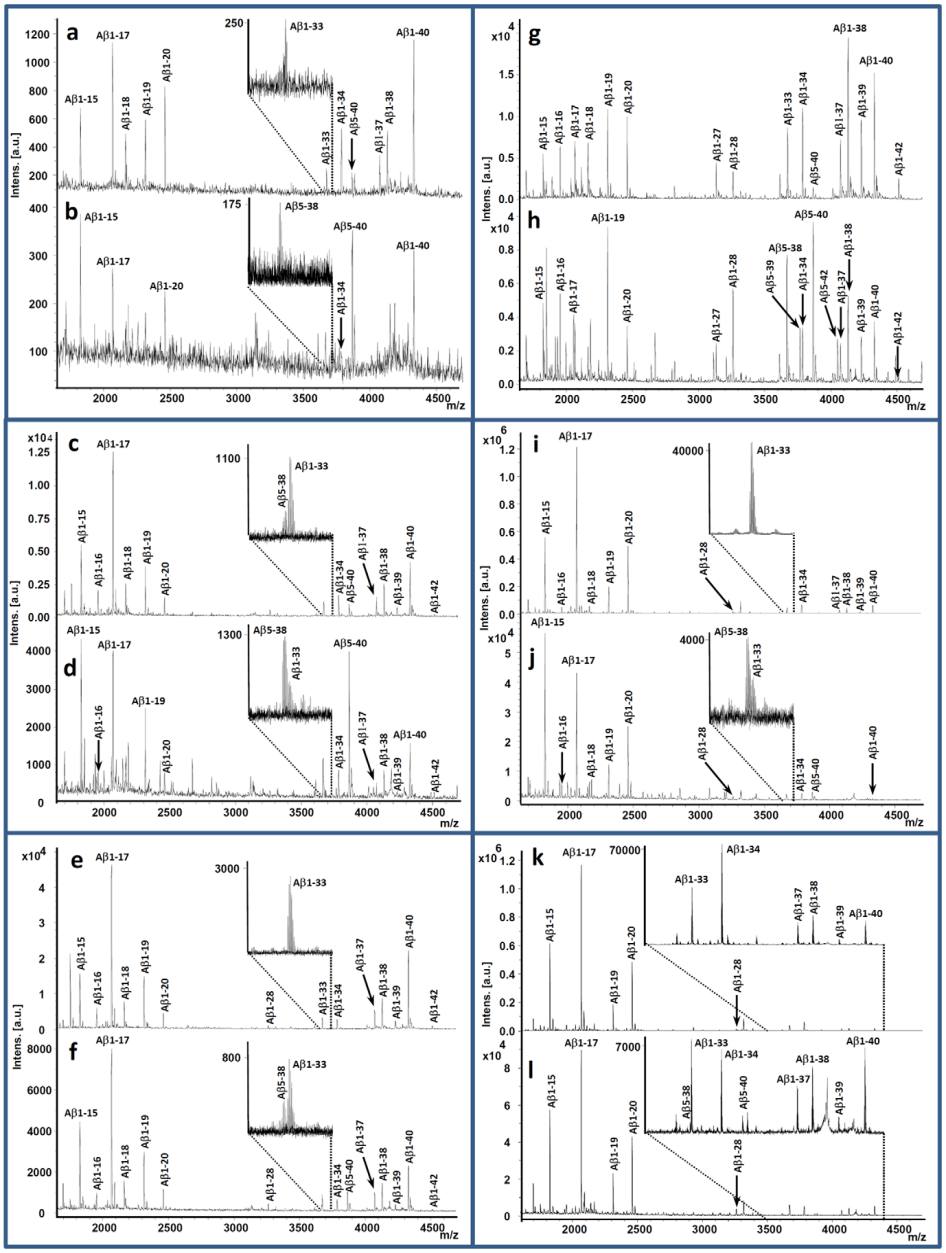
Mass spectra of Aβ isoform patterns in all cell models investigated. SH-SY5Y APP695wt cells treated with DMSO (Panels a and c), 5 µM β-secretase inhibitor IV (Panel b) or 10 µM AZ-20 (Panel d). SH-SY5Y APP695swe cells treated with DMSO (Panel e) or 10 µM AZ-20 (Panel f). 7PA2 APP751 V717F cells treated with DMSO (Panel g) or 10 µM AZ-20 (Panel h). HeLa APPswe cells treated with DMSO (Panel i) or 10 µM β-secretase inhibitor IV (Panel j). HeLa APPswe scrambled siRNA transfected control cells (Panel k) and cells transfected with single oligo siRNAs against BACE1 (Panel l). The mass-to-charge ratio (m/z) of the [M+H]^+^ ion of Aβ5-38 is very close to that of Aβ1-33, causing the peaks to partially overlap and making quantification difficult, wherefore both isoforms were excluded from quantitative analysis. Those peptides are instead presented in these mass spectra as expanded inserts (except for panels g-h where they are clearly visible).

**Figure 2:**
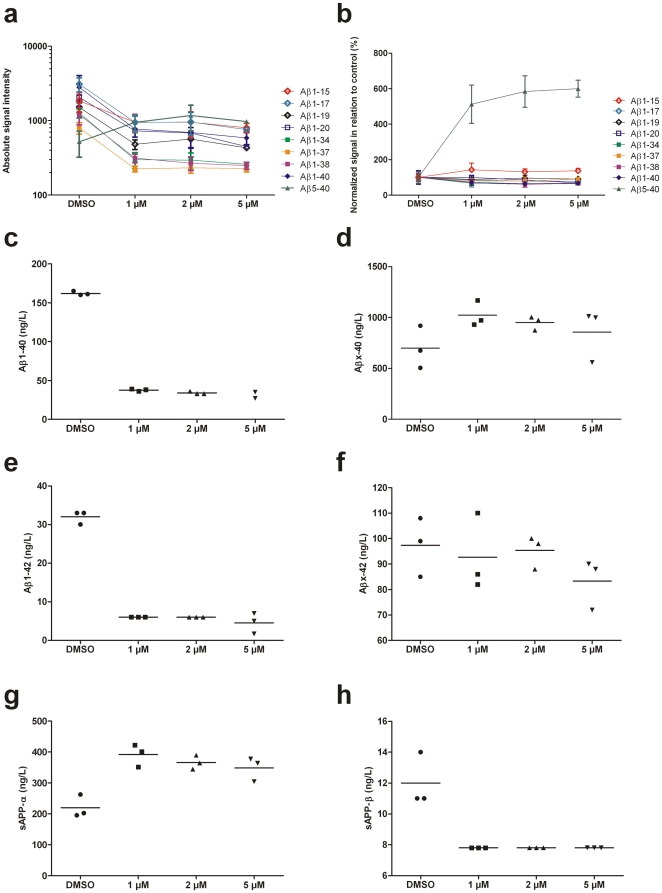
SH-SY5Y APP695wt cells treated with β-secretase inhibitor IV. Peak intensities of all Aβ isoforms detected (Panel a). Normalized (against the sum for all the Aβ peaks in the spectrum) levels in relation to controls (DMSO) (Panel b). Immunoassay measurements of Aβ1-40 and AβX-40 (Panels c–e), Aβ1-42 and AβX-42 (Panels f–g), sAPP-α and sAPP-β (Panels h–i). Note that the X-40/42 groups may consist of a large variety of species not detected with the MALDI-TOF-MS method utilized in this work, including such spanning the β-secretase site. N = 3 for each concentration. Data are means and error bars are SD in panels a–b.

To investigate the effects of BACE1-inhibition on neuronally secreted Aβ, human neuroblastoma SH-SY5Y cells stably expressing human APP695wt were treated with the BACE1 inhibitor β-secretase inhibitor IV. Immunoprecipitation-mass spectrometry (IP-MS) analysis of the cell media displayed a distinct shift in the Aβ isoform pattern in response to treatment including an anticipated decrease in the peak intensity of Aβ1-40 but increased intensities of Aβ5-38 and Aβ5-40 (Fig. 1a–b, Fig. 2a). Relative to other isoforms, treatment clearly increased the levels of Aβ5-40, while the levels of most other isoforms tended to be reduced (Fig. 2b). These BACE1 induced alterations in the Aβ isoform pattern were supported by immunoassay data showing decreased concentrations of Aβ1-40 and Aβ1-42 but no major effects on AβX-40 and AβX-42 (Fig. 2c–f). The concentrations of sAPP-β decreased and sAPP-α increased in response to treatment, further supporting that BACE1 inhibition induces a shift in APP processing pathways (Fig. 2g–h). The altered Aβ peptide pattern was unique to BACE1 inhibition and was not seen when cells were treated with a γ-secretase inhibitor or a cathepsin B-inhibitor (Fig. S1).

### The effects of BACE1 inhibition are consistent across cell models and treatments

**Figure 3:**
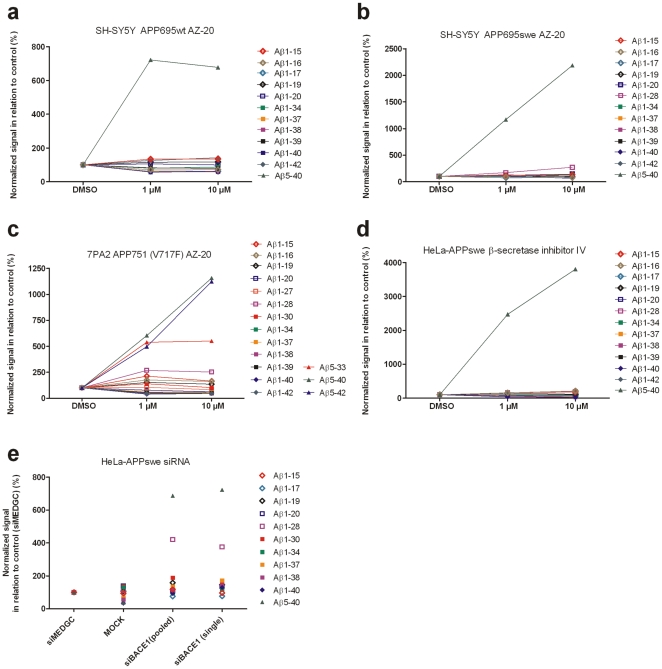
Cell medium Aβ isoform patterns. SH-SY5Y APP695wt cells treated with AZ-20 (Panel a). SHSY-5Y APP695swe cells treated with AZ-20 (Panel b). 7PA2 APP751 V717F cells treated with AZ-20 (Panel c). HeLa-APPswe cells treated with β-secretase inhibitor IV (Panel d). HeLa-APPswe mock and scrambled siRNA-transfected control cells, and cells transfected with single oligo siRNA or pooled siRNA against BACE1 (Panels e). N = 1 for each concentration and treatment.

We evaluated if the effect on secreted Aβ peptides was compound-specific or a general response to BACE1 inhibition. SH-SY5Y APP695wt cells were treated with another BACE1-inhibitor, the potent AstraZeneca compound AZ-20 [21] (IC_50_ = 8.2±1.4 nM [SEM], N = 3), which had very similar effects as β-secretase inhibitor IV on the Aβ isoform pattern, including concentration-dependent increases in the relative levels of Aβ5-38 and Aβ5-40 (Fig. 1c–d, Fig. 3a, Fig. S2a). We also analyzed the effects of AZ-20 on SH-SY5Y cells transfected with the Swedish APP mutation (APP695swe). Again AZ-20 increased the relative levels of Aβ5-38 and Aβ5-40 (Fig. 1e–f, Fig. 3b). To examine if the effects of BACE1 inhibition were restricted to neuronal cells, a CHO-cell line (7PA2 APP751 with the V717F FAD-mutation) was treated with AZ-20. These cells released a complex Aβ isoform pattern (Fig. 1g–h), but treatment increased the relative levels of Aβ5-33, Aβ5-38, Aβ5-40, and Aβ5-42 (Fig. 1.g-h, Fig. 3c, Fig. S2c).

To further verify that the effects on Aβ secretion were general across different cell models, we treated HeLa-APPswe cells with β-secretase inhibitor IV, resulting in increased relative levels of Aβ5-38 and Aβ5-40 (Fig. 1i–j, Fig. 3d, Fig. S2d–e). Similar results were obtained for siRNA against BACE1 in HeLa-APPswe cells (Fig. 1.k–l, Fig. 3e, Fig. S2e).

Together, these experiments indicated that the general effect of BACE1 inhibition was to reduce the release of most Aβ peptides, but increase the release of Aβ5-38 and Aβ5-40.

### BACE1 inhibition affects CSF Aβ peptides in mammals

**Figure 4:**
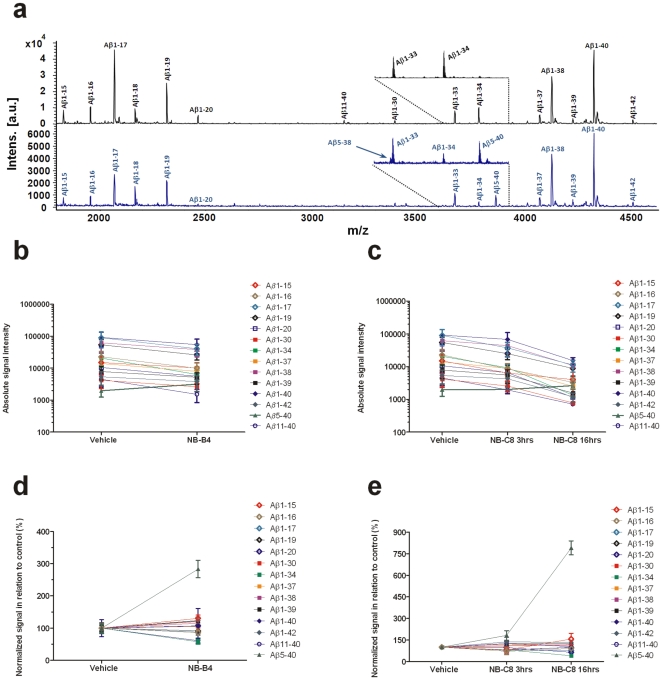
CSF Aβ isoform patterns. Dogs treated with vehicle (N = 15), NB-C8 (N = 3 at 3 hours, N = 3 at 16 hours) and NB-B4 (N = 5 at 6 hours). Mass spectra of the Aβ isoform pattern from dogs treated with placebo (Panel a, upper panel) or NB-C8 16 hours post treatment (Panel a, lower panel). The expanded sections show Aβ1-33, Aβ1-34, Aβ5-38 and Aβ5-40. Aβ1-33 and Aβ5-38 were excluded from quantitative analysis since their peaks partially overlap, making quantification difficult. Absolute (Panels b–c) and normalized (Panels d–e) mass spectral peak intensities of all detected Aβ isoforms. Statistical significances were tested for normalized peak intensities comparing different treatments. For NB-B4, significant differences were seen for Aβ5-40 (P = 0.001), Aβ1-34 (P = 0.001) and Aβ11-40 (P = 0.002). For NB-C8, significant differences were seen between vehicle and treatment at 16 h for Aβ5-40 (P = 0.01) and Aβ1-34 (P = 0.05). Data are means; error bars are SD.

We proceeded to verify that the altered neuronal Aβ pattern may be used to identify biochemical effects of BACE1-inhibition in CNS in mammals, by examining CSF from dogs treated with two different BACE1 inhibitors (the cyclic sulfoxide hydroxyethylamine NB-B4 and the oxazine derivative NB-C8, obtained from Novartis [22]). In total, 14 Aβ isoforms were reproducibly detected by IP-MS in all dogs and used for further analysis (Fig. 4a). Treatment with either of the compounds reduced the absolute CSF signal of all Aβ isoforms except Aβ5-40 (Fig. 4b–c). For relative levels, a reduction was seen for Aβ1-34, while Aβ5-40 clearly increased (Fig. 4d–e). For NB-C8, multiple time-points where analyzed and the relative levels of Aβ5-40 increased over time (Fig. 4e). Treatment decreased the absolute, but not the relative levels of Aβ1-40 (Fig. 4b–e).

### The CSF Aβ peptide pattern as a pharmacodynamic fingerprint of BACE1 inhibition

**Figure 5:**
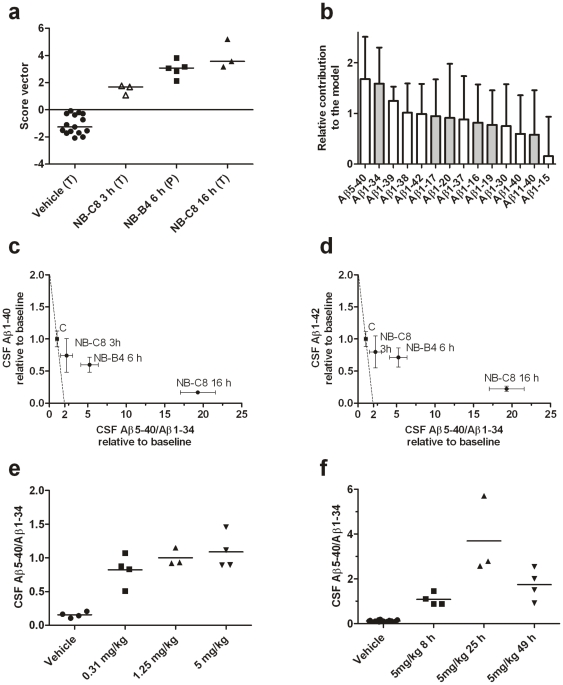
Multivariate analysis and CSF Aβ5-40/Aβ1-34. Multivariate discriminant analysis of the CSF Aβ pattern for treatment compared with placebo in dogs treated with vehicle (N = 15), NB-C8 (N = 3 at 3 h, N = 3 at 16 h) and NB-B4 (N = 5 at 6 h) (Panels a–b). Score vector results including medians (Panel a). Samples were taken 3 h (open triangles), 6 h (filled squares) or 16 h (filled triangles) after treatment. The groups denoted by “T” were used for constructing the multivariate model while “P” represents the prediction set used for testing the stability of the model. In the construction of the model, all dogs treated with NB-C8 were regarded equal, independent of time after drug administration. Relative contributions of different isoforms to group separations, with increased or decreased relative levels in the treated groups, are shown by white or grey bars, respectively (Panel b). Change of the CSF Aβ5-40/Aβ1-34 ratio in relation to change of CSF Aβ1-40 (Panel c) and CSF Aβ1-42 (Panel d). The Aβ5-40/Aβ1-34 ratio was a more sensitive biomarker than Aβ1-40 and Aβ1-42 in terms of change from baseline (the dotted lines indicate predicted correlations for biomarkers affected equally by treatment). Dogs treated with vehicle (N = 4) and BACE1 inhibitor S obtained from Janssen (N = 4 for each dosage) (Panel e). The CSF Aβ5-40/Aβ1-34 ratio completely separated dogs on active treatment versus placebo (P = 0.005 using the Mann-Whitney U test for comparison of all animals on active treatment versus placebo). Time-dependent dynamics of the ratio in high dose treatment (Panel f).

To investigate if a combination of Aβ peptides could be used to increase the separation between groups, we performed a multivariate analysis including all identified Aβ isoforms, and compared dogs on active treatment and placebo for each investigated drug. It was possible to construct a model for NB-C8 versus placebo that achieved a complete separation between groups that increased with time after drug administration (Fig. 5a). The time dependency was still present when data from the NB-B4 treated dogs were entered into the model. The most important Aβ peptides in the model were Aβ5-40 with increased relative intensity and Aβ1-34 with decreased relative intensity after treatment (Fig. 5b). The CSF Aβ5-40/Aβ1-34 ratio was a more sensitive measurement of BACE1 inhibition than the expected pharmacodynamic markers CSF Aβ1-40 and CSF Aβ1-42 (Fig. 5c–d). It should be noted that this study was carried out on post-mortem CSF. To verify that the changed Aβ pattern was not a result of unspecific degradation in post-mortem CSF or cell media, and to further validate the generalizability of the CSF biomarker fingerprint across different BACE1-inhibitors, we evaluated CSF Aβ5-40 and Aβ1-34 in an independent group of live dogs treated with another BACE1-inhibitor (BACE1-inhibitor S, obtained by Janssen). The CSF Aβ5-40/Aβ1-34 ratio increased in dogs on active treatment versus dogs treated with placebo (Fig. 5e). For the highest treatment dose we also evaluated temporal effects, and noted maximal effects on the CSF Aβ5-40/Aβ1-34 ratio at 25 h after treatment and a regression towards baseline at 49 h in this single-dose study (Fig. 5f).

## Discussion

BACE1 is one of the prime targets for disease-modifying AD therapy [9], [23]–[27], and BACE1 inhibition has also been suggested as a therapeutic approach for nerve injury [28]. Here we show that BACE1-inhibition induces a specific CSF Aβ pattern, primarily characterized by reduced relative levels of Aβ1-34 and increased relative levels of Aβ5-40. In addition to measuring single Aβ isoforms, including Aβ1-40 and Aβ1-42 by enzyme-linked immunosorbent assays (ELISAs) or other immunoassays, the CSF Aβ pattern (including the Aβ5-40/Aβ1-34 ratio) may give complementary information on the change in Aβ and APP metabolism. We propose that these biomarkers are a useful pharmacodynamic fingerprint of BACE1 inhibition to detect *in vivo* drug effects in the CNS, which may accelerate drug development, by verifying compound efficiency in the target organ, identifying treatment responders and facilitating optimal dosage. We verified the generalizability of the biomarker pattern across multiple cell types and in dog CSF, using several different BACE1-inhibiting regimes. Albeit with some differences for individual models, the BACE1 inhibition biomarker panel was generally consistent, especially regarding the increase in Aβ5-40 after BACE1 inhibition, which was a ubiquitous finding. The decrease in Aβ1-34 was prominent in CSF but not in all cell models. Humans and dogs have identical Aβ sequences and very similar CSF Aβ patterns. γ-secretase inhibition induces a unique CSF Aβ peptide pattern in both species [29], [30], supporting dog models for studies on CSF pharmacodynamic biomarkers.

**Figure 6:**
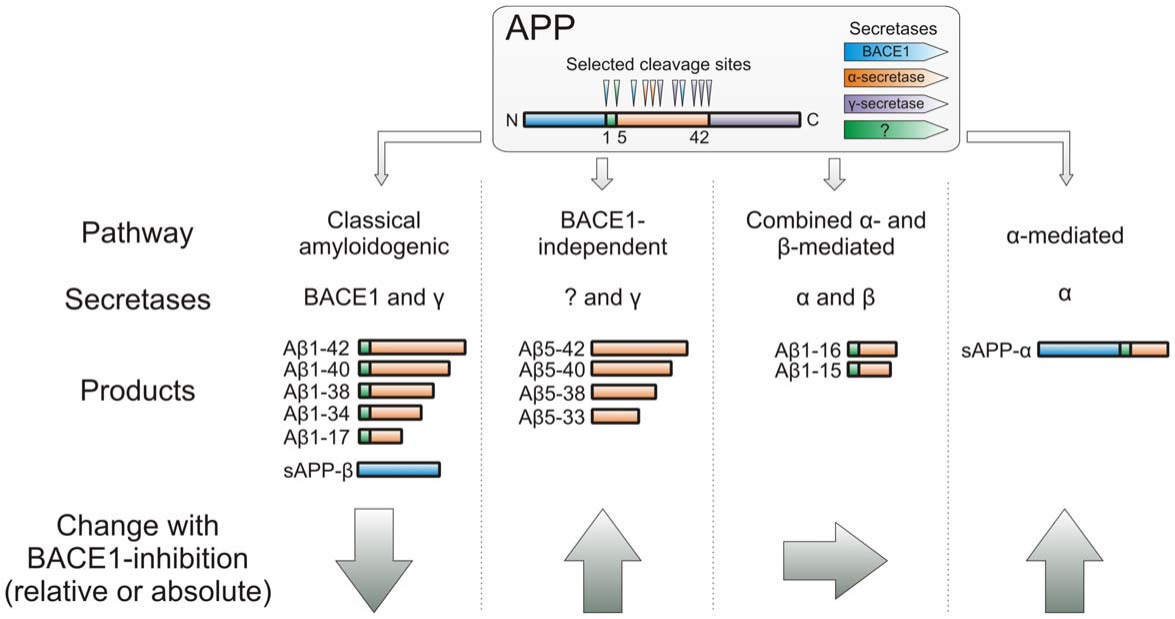
Summary of processing pathways. The main pathways of Aβ peptide release and how these are affected by BACE1 inhibition (arrows indicate absolute and/or relative changes). The APP box shows major APP cleaving secretases with selected cleavage sites that depend on them.

As expected, we also found decreased levels of Aβ1-40 and Aβ1-42, in accordance with previous observations in a rhesus monkey model [31] and in humans [32]. Yet, CSF Aβ1-42 may not be an optimal biomarker in AD trials. For example, γ-secretase inhibition in AD patients lack effects on CSF Aβ1-40 and Aβ1-42 [33], which might suggest that the tolerated doses in humans were too low to produce the anticipated reductions of CSF Aβ1-40 and Aβ1-42. However, γ-secretase inhibition reduces the levels of CSF Aβ1-34 and increases the levels of CSF Aβ1-15 and Aβ1-16 even at doses not effecting Aβ1-40 and Aβ1-42, indicating that other isoforms may be more sensitive pharmacodynamic markers than Aβ1-40 and Aβ1-42 [18], . Aβ1-34 is an intriguing peptide, since both the cleavages at position 1 and 34 depend on BACE1, and the cleavage at position 34 also depends on γ-secretase [35], [36]. Together, these studies illustrate the complexity of therapy-altered APP catabolomics (Fig. 6).

Much is known about *in vitro* properties of BACE1 inhibitors [37], but there is a need for characterization of their effects *in vivo*. Our data fully support BACE1 as the main β-secretase for full length Aβ peptides [38]–[40], but also point to an alternative APP processing pathway, producing Aβ5-X peptides. The increased levels of Aβ5-X in cell media and CSF after BACE1 inhibition is consistent with previous reports on Aβ5-40 formation being stable despite BACE1 inhibition [17], [41], and might be linked to caspase activity [41]. It is not clear to what extent BACE1 and the unidentified enzyme cutting at position 5 in the Aβ sequence compete for the same APP substrates. Further studies on APP compartmentalization and the molecular characteristics of the Aβ5-X isoforms, including their resistance to degradation, are necessary to clarify how this novel pathway relates to the established APP processing pathways. It remains to be determined if the concentration of Aβ5-X isoforms varies with cerebral BACE1 activity, which may be increased in AD [27], [42]–[47]. Small amounts of Aβ5-X peptides are present in AD brains [13], [48], [49] (and less in control brains [14]), in vascular lesions with amyloid angiopathy and in neurofibrillary tangles [41]. Studies of the biological functions of Aβ5-X may give clues on the role of this novel pathway of APP degradation, and reveal if its regulation participate in AD pathogenesis.

In conclusion, CSF Aβ peptide levels seem to be direct measurements of the effects of BACE1 inhibition on APP-processing in the CNS, and the strongest effects are seen for Aβ5-40 and Aβ1-34. The CSF Aβ5-40/Aβ1-34 ratio is a sensitive acute pharmacodynamic biomarker for BACE1 inhibition. Studies on the APP processing pathway generating Aβ5-40, and identification of the enzyme(s) responsible for its production, may aid to the current understanding on how APP and Aβ metabolism exerts its putative damaging effects in AD.

## Materials and Methods

### Ethics Statement

This study was carried out with ethical approvals given by the Basel City Cantonal Veterinary Authority (Novartis study) and the Ethical Committee on Laboratory Animal Testing (ECD, Janssen Beerse, Janssen study, Permit number: 2009-252-SD).

### Cell experiments

SH-SY5Y cells [50] obtained from the European Collection of Cell Cultures (ECACC 94030304) (stably expressing human APP695wt or APPswe), 7PA2 cells obtained from the Laboratory of Dennis J. Selkoe at Harvard Medical School, Boston (APP751 with the V717F mutation [51], [52]), and HeLa cells [53] (APPswe), were maintained in Dulbecco's modified Eagle's medium F-12 (Invitrogen) supplemented with 10% fetal bovine serum, L-glutamine and antibiotics. SH-SY5Y cells were treated with the BACE1-inhibitor β-secretase inhibitor IV (Calbiochem, Merck, compound 3 in [54]) or DMSO and incubated for 20 h. SH-SY5Y cells and 7PA2 cells were treated with the BACE1 inhibitor AZ-20 [21] or DMSO and incubated for 16 h. HeLa cells were treated with β-secretase inhibitor IV or DMSO and incubated for 4 h. Two Stealth siRNAs against BACE1 (Invitrogen) were used for reverse transfection in HeLa-APPswe cells. A scrambled siRNA sequence containing medium GC content was used as a control. Sixty-nine h after reverse transfection using Oligofectamine, the medium was replenished with medium containing 10% Alamar Blue, and incubated for 3 h. All cell medium was stored at −80°C.

### Animal experiments

The BACE1 inhibitors NB-B4 and NB-C8 (obtained from Novartis [22]) were used in experiments with 26 beagle dogs, aged 2–11 years. Animals were pseudorandomly allocated to groups to control for the effects of age (NB-B4 and NB-C8) and gender (NB-C8 only). A vehicle group was included. Fasting animals were dosed orally with 2 mL/kg (NB-B4) or 1.5 mL/kg (NB-C8) (in 0.5% methylcellulose/0.1% Tween80) and sacrificed with intravenous pentobarbital overdose (at least 50 mg/kg, approximately 0.5 mL/kg), at 6 h (NB-B4), 3 h (NB-C8) or 16 h (NB-C8). CSF samples were taken from the cisterna magna, aliquoted and stored at −80°C.

The BACE1-inhibitor S (obtained from Janssen) was used in an experiment with 16 beagle dogs, aged 1–4 years. Two male and two female dogs were included per group. A vehicle group was included. Fasting animals were dosed orally with 1 mL/kg BACE1-inhibitor S at doses 0.31, 1.25 and 5 mg/kg (in 20% hydroxypropyl-β-cyclodextrin and tween). CSF samples were taken from the cisterna magna under short general anesthesia (0.2 mL medetomidine and 2 mL propofol), aliquoted and stored at −80°C.

### Immunoassays

Cell medium was analyzed for sAPP-α and sAPP-β using the MSD sAPPα/sAPPβ Multiplex Assay [27] (Meso Scale Discovery, Gaithersburg, MD, USA) and for Aβ1-40/42 and AβX-40/42 using the Luminex xMAP assay INNO-BIA Aβ forms [55] (Innogenetics, Gent, Belgium). Cells treated with AZ-20 were assayed for Aβ40 using ELISA (Invitrogen) to determine the half maximal inhibitory concentration (IC_50_).

### Immunoprecipitation and mass spectrometry

Aβ peptides were immunoprecipitated using Aβ-specific antibodies coupled to magnetic beads [15]. Briefly, 4 µg of the anti-Aβ antibodies 6E10 and 4G8 (Signet Laboratories, Dedham, MA, USA) was separately added to 50 µL each of magnetic Dynabeads M-280 Sheep Anti-Mouse IgG (Invitrogen, Carlsbad, CA, USA). The 6E10 and 4G8 antibody-coated beads were mixed and added to the cell media or CSF to which 0.025% Tween20 in phosphate-buffered saline (pH 7.4) had been added. After washing, using the KingFisher magnetic particle processor, the Aβ isoforms were eluted using 100 µL 0.5% FA. Mass spectrometry measurements were performed using a Bruker Daltonics UltraFleXtreme matrix-assisted-laser-desorption/ionization time-of-flight/time-of-flight (MALDI TOF/TOF) instrument or a Bruker Daltonics AutoFlex MALDI TOF (Bruker Daltonics, Bremen, Germany). All samples were analyzed in duplicate. An in-house MATLAB (Mathworks Inc. Natick, MA, USA) program was used for relative quantification of the Aβ isoforms. For each peak the sum of the heights for the three highest isotopes were averaged followed by normalization against the sum for all the Aβ peaks in the spectrum. When a ratio between two Aβ isoforms was calculated the normalization step was omitted. It should be noted that a relative quantification cannot be interpreted as a direct reflection of an absolute or relative abundance of an isoform since the ionization efficiency might be different for different isoforms and since different isoforms are more hydrophobic than others.

### Liquid chromatography and tandem mass spectrometry

To confirm isoform identities, the immunoprecipitates were also analyzed by liquid chromatography (LC) combined with high resolution tandem mass spectrometry (MS-MS) [15], on an Ettan MDLC nanoflow chromatographic system (GE Healthcare) using HotSep Kromasil C4 columns (G&T Septech) coupled to a Thermo LTQ-FT Ultra electrospray ionization hybrid linear quadrupole ion trap/Fourier transform ion cyclotron resonance (ESI-LQIT/FTICR) mass spectrometer (Thermo Fisher Scientific). All spectra were acquired in FTICR mode and collision induced dissociation was used to obtain fragment ion data.

### Statistical analysis

In the dog experiments, we compared each mass spectrometric peak between different treatment groups using the non-parametric Mann-Whitney U test (corrected for multiple comparisons using Bonferroni correction) for pair-wise comparisons and the Kruskal-Wallis test (followed by Dunn's post hoc test) for multiple groups. Multivariate analysis was performed using the orthogonal projections to latent structures discriminant analysis (OPLS-DA) algorithm [56], [57] (SIMCA-P+, v.12, Umetrics, Umeå, Sweden), which finds the direction in the multivariate space spanned by the analytes which best separates the predefined groups. The studies on dogs treated with NB-B4 and NB-C8 were conducted separately, with individual vehicle groups for each, but no differences in biomarker levels were found between the two vehicle groups, wherefore all vehicle treated animals were used as one control group in this analysis.

## Supporting Information

Figure S1 **Mass spectra displaying the Aβ isoform pattern in media from SH-SY5Y APP695wt cells treated with DMSO (Panel a), a cathepsin-B inhibitor (Z-FA-FMK 5 µM) (Panel b), and γ-secretase inhibitor (DAPT 1 µM) (Panel C).**
(TIF)

Figure S2 **Absolute spectral peak intensities of all Aβ isoforms detected in cell media.** SH-SY5Y APP695wt cells treated with AZ-20 (Panel a). SH-SY5Y APP695swe cells treated with AZ-20 (Panel b). 7PA2 APP751 V717F cells treated with AZ-20 (Panel c). HeLA-APPswe cells treated with β-secretase inhibitor IV (Panel d). HeLA-APPswe mock and siMEDGC transfected control cells, and cells transfected with single oligo SiRNA or pooled SiRNA (Panel e).
(TIF)
