# Supplementary figures and images for: BACE1 Inhibition Induces a Specific Cerebrospinal Fluid β-Amyloid Pattern That Identifies Drug Effects in the Central Nervous System
Source: PLoS One. 2012 Feb 6;7(2):e31084. doi: 10.1371/journal.pone.0031084 (PMC3273469; doi:10.1371/journal.pone.0031084)

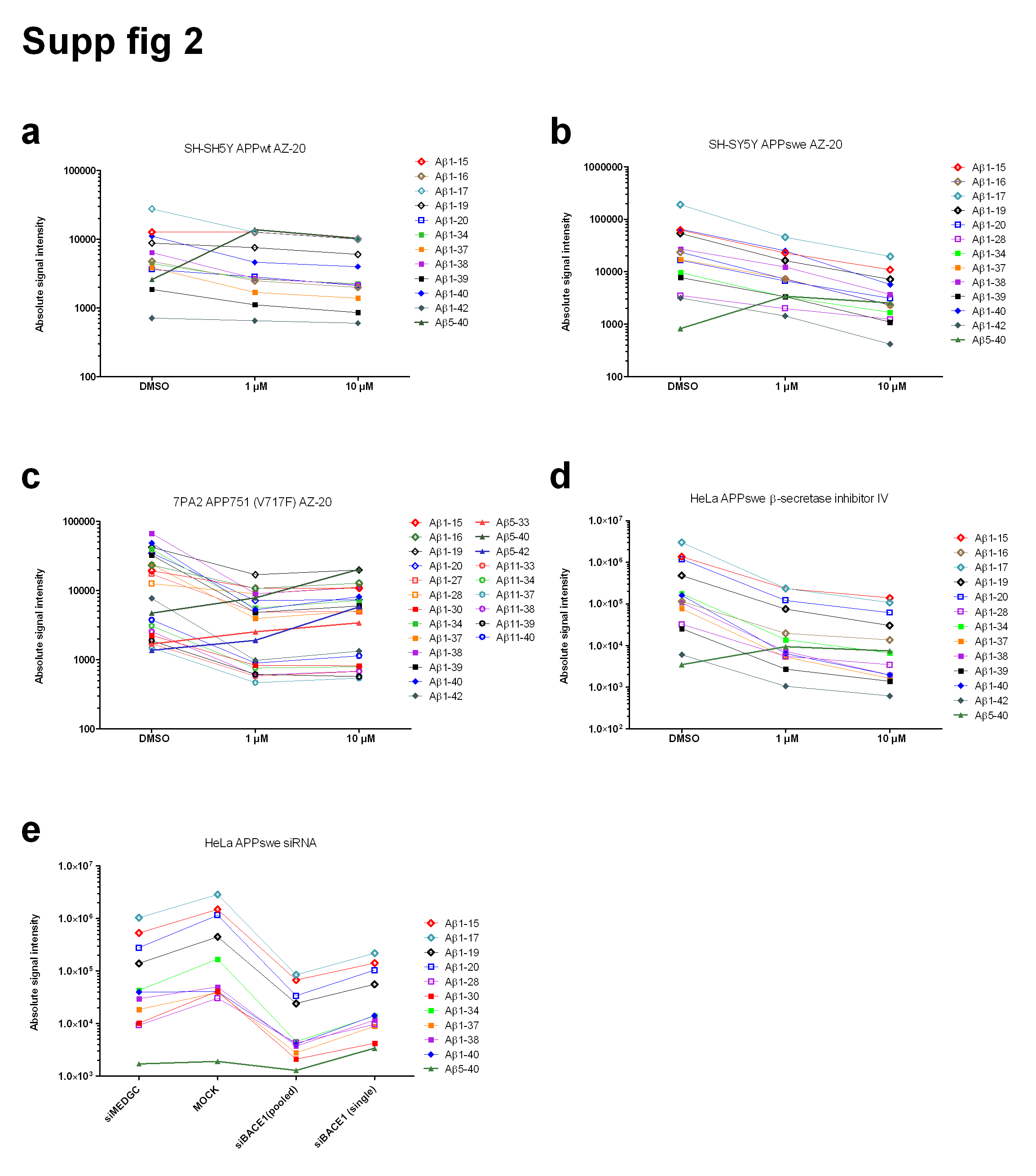

Supplement: Figure S2 — Absolute spectral peak intensities of all Aβ isoforms detected in cell media. SH-SY5Y APP695wt cells treated with AZ-20 (Panel a). SH-SY5Y APP695swe cells treated with AZ-20 (Panel b). 7PA2 APP751 V717F cells treated with AZ-20 (Panel c). HeLA-APPswe cells treated with β-secretase inhibitor IV (Panel d). HeLA-APPswe mock and siMEDGC transfected control cells, and cells transfected with single oligo SiRNA or pooled SiRNA (Panel e). (TIF) [file pone.0031084.s002.tif]
